# Postconditioning promotes recovery in the neurovascular unit after stroke

**DOI:** 10.3389/fncel.2023.1260389

**Published:** 2023-09-08

**Authors:** Elga Esposito, Ester Licastro, Ornella Cuomo, Eng H. Lo, Kazuhide Hayakawa, Giuseppe Pignataro

**Affiliations:** ^1^Neuroprotection Research Laboratories, Department of Radiology, Massachusetts General Hospital and Harvard Medical School, Charlestown, MA, United States; ^2^ Consortium International pour la Recherche Circadienne sur l'AVC (CIRCA); ^3^Division of Pharmacology, Department of Neuroscience, Reproductive and Dentistry Sciences, School of Medicine, University of Naples Federico II, Naples, Italy

**Keywords:** stroke, ischemic postcoditioning, astrocytes, neuroprotecion, angiogenesis, neurogenesis, BDNF

## Abstract

**Background and purpose:**

Experimental studies suggest that ischemic postconditioning interferes with cell death mechanisms and reduces infarction during the acute phase after focal cerebral ischemia. Postconditioning may be a practically feasible way to promote stroke recovery, but many drawbacks prevent its clinical translation. First, all existing studies are mostly on acute 24 h outcomes. Second, the mechanisms of protection and augmented long-term benefits remain unclear. Our study aims to define some of the mechanisms that explain long-term benefits of improved recovery.

**Methods:**

Male Sprague–Dawley rats were subjected to 100-min transient middle cerebral artery occlusion (MCAO) or postconditioning (100-min middle cerebral artery occlusion plus 10-min reperfusion plus 10-min reocclusion). After 3 days or 2 weeks, infarct volumes, western blot, and immunohistochemical markers of neurogenesis and angiogenesis were quantified. Fluorocitrate (FC) or saline were administrated ICV (intraventricular injection) every other day starting on day 5 after focal cerebral ischemia, animals were recovered for 2 weeks.

**Results:**

After postconditioning BDNF protein expression levels increased compared to animals subjected to MCAO. Immunostaining showed that BDNF increased specifically in astrocytes. Moreover, when astrocytes were metabolically inhibited by fluorocitrate the postconditioning neuroprotective effect together with the postconditioning-dependent new angiogenesis and neurogenesis, were no longer observed.

**Conclusion:**

These results suggest for the first time that therapeutic effects of postconditioning may involve the promotion of neurogenesis and angiogenic remodeling, via BDNF released by astrocytes, during the recovery phase after focal cerebral ischemia.

## Introduction

Postconditioning represents a translationally exciting and relevant approach for cerebrovascular disease. The neuroprotection induced by early reperfusion interruption is probably associated with changes in cerebral blood flow. Subsequent events as free radical production, BBB integrity, inflammation and endothelial function have also been shown to be involved (Gao et al., 2008; Zhao, 2011; Zhao et al., 2012; Joo et al., 2013). The majority of experimental studies have focused on the acute mechanisms of ischemic postconditioning. The effect of ischemic postconditioning in brain for longer periods post-stroke remains to be fully elucidated. In a previous study we suggested that the therapeutic effects of postconditioning may involve the promotion of neurogenesis and angiogenic remodeling during the recovery phase after focal cerebral ischemia (Esposito et al., 2015). However, mechanisms for long-term outcomes and delayed stroke recovery remain unclear.

After a National Institutes of Health workshop in 2001, where the concept of neurovascular unit (NVU) was first proposed (Lo et al., 2003), CNS injury and disease are now well known to be the result from the dysfunction and death not only of neurons but of signaling among different cell types. Together these cell types, including astrocytes, pericytes, smooth muscle cells, endothelial cells, oligodendrocytes, microglia and neural and glial precursor cells, make up the neurovascular unit. Astrocytes comprise the major of non-neuronal cell population in the mammalian neurovascular unit and can produce many factors for protecting and restoring neurons (Tiedt et al., 2022).

Interestingly, astrocytes normally express BDNF, although at lesser levels than neurons (Saha et al., 2006; Giralt et al., 2010; Fulmer et al., 2014; Hong et al., 2016; de Pins et al., 2019). BDNF is known to increase with oxidative stress occurring during ischemia–reperfusion (I/R) injury (Qin et al., 2022) as part of an antioxidant defense and it has been previously showed that postconditioning stimulus can increase BDNF (Wu et al., 2015). Moreover, BDNF can stimulate neurogenesis (Schäbitz et al., 2007) and angiogenesis (Kermani and Hempstead, 2007).

Our current study tests the hypothesis that postconditioning affects astrocyte signaling via BDNF in order to promote neurovascular remodeling. Our results support this hypothesis by showing that after using fluorocitrate, an astrocyte inhibitor, the postconditioning neuroprotective effect together with the postconditioning dependent new angiogenesis and neurogenesis, were no longer observed.

## Methods

### Middle cerebral artery occlusion and ischemic postconditioning

All experiments were performed following protocols approved by Massachusetts General Hospital Institutional Animal Care and Use Committee in accordance with the National Institute of Health Guide for the Care and Use of Laboratory Animals. Adult male Sprague–Dawley rats (Charles River) weighting 320–340 g were anesthetized with isoflurane (1.5%) in 30%/70% oxygen/nitrous oxide. Transient focal ischemia was induced by introducing a 5-O surgical monofilament nylon suture into the middle cerebral artery (MCA) (Doccol) for 100 min. Ischemic postconditioning was induced as previously described (Pignataro et al., 2011). Briefly, after 100 min of occlusion, reperfusion was established for 10 min after which the MCA was reoccluded for another 10 min. To analyze cell renewal processes, BrdU was dissolved in PBS at 10 mg/ml and injected intraperitoneally to a dose of 50 mg/kg every second day for 2 weeks. To demonstrate that during the recovery phase after stroke, astrocytes are involved in neuroprotection induced by postconditioning, astrocytes were metabolically inhibited with fluorocitrate (FC). FC (40 nmol) or saline were administrated ICV (intraventricular injection) every other day starting on day 5 after focal cerebral ischemia. Animals were recovered for 2 weeks. All procedures and measurements were performed in a blinded and randomized fashion.

### Histology and immunohistochemistry

Animals were euthanized 2 weeks after ischemic onset. Brains were quickly removed and frozen, and coronal sections of 20 μm thickness were prepared. Infarction volumes were quantified on Nissl-stained sections using the “indirect” morphometric method with Image J software. Immunohistochemistry was performed as described before (Esposito et al., 2013) using primary antibodies anti-Doublecortin (DCX) (1:100, #18723 Abcam) and anti- BrdU (1:200, B35130 Invitrogen) as a marker of neurogenesis, and anti-type IV Collagen (1,10, #1340–01 SouthernBiotech) for vascular remodeling and anti-Ki67 (1,500, #1667 Abcam), as proliferation marker, for detecting angiogenesis. GFAP (1,200, #130300 Invitrogen), as a marker of astrocytes, PDGFβ (1,200, #AF1042 R&D systems), as marker of pericytes, Iba1(1,200, #019–19,741 WAKO or Abcam 5,076), as marker of microglia, or NeuN (1,200, #MAB377 Millipore) as marker of neurons, were co-stained with BDNF (1,200, # ab46176 Abcam) or VEGF (1,50, #sc152 Santa Cruz). Three nonoverlapping areas (0.125 mm^2^ per area) were chosen in the boundary zone of the ischemic core to analyze the peri-infarct area.

### Western blot analysis

Western blot was performed as previously reported (Esposito et al., 2020). Cell Lysis Buffer (Cell signaling) plus protease inhibitors was added to the brain sample (100 mg of tissue to 1 ml of buffer). Each sample was loaded onto 4–20% Tris-glycine gels. After electrophoresis and transferring to nitrocellulose membranes, the membranes were blocked in Tris-buffered saline containing 0.1% Tween 20 and 0.2% I-block (Tropix, T2015) for 90 min at room temperature. Membranes were then incubated overnight at 4°C with the following primary antibodies, anti-β -actin (1:1,000, #A5441 Sigma-Aldrich), anti-BDNF (1:500, #Y075023 Abcam), anti-VEGF (1:500, #SC1822 Santa Cruz), anti-MMP2 (1:500, #IM33L Oncogene) and anti-MMP9 (1:500, MAB13421 Chemicon). After incubation with peroxidase-conjugated secondary antibodies, visualization was enhanced by chemiluminescence (GE Healthcare, NA931- anti-mouse, or NA934- anti-rabbit). Optical density was assessed using the NIH ImageJ analysis software.

### Statistical analysis

Values are expressed as mean ± sd. Multiple comparisons were evaluated by one-way ANOVA followed by Tukey’s test. *p-*values of *p* < 0.05 were considered statistically significant. Statistical analyses were performed using GraphPad Prism 6.

## Results

### Ischemic postconditioning increases BDNF and MMP9 protein levels after stroke

**Figure 1:**
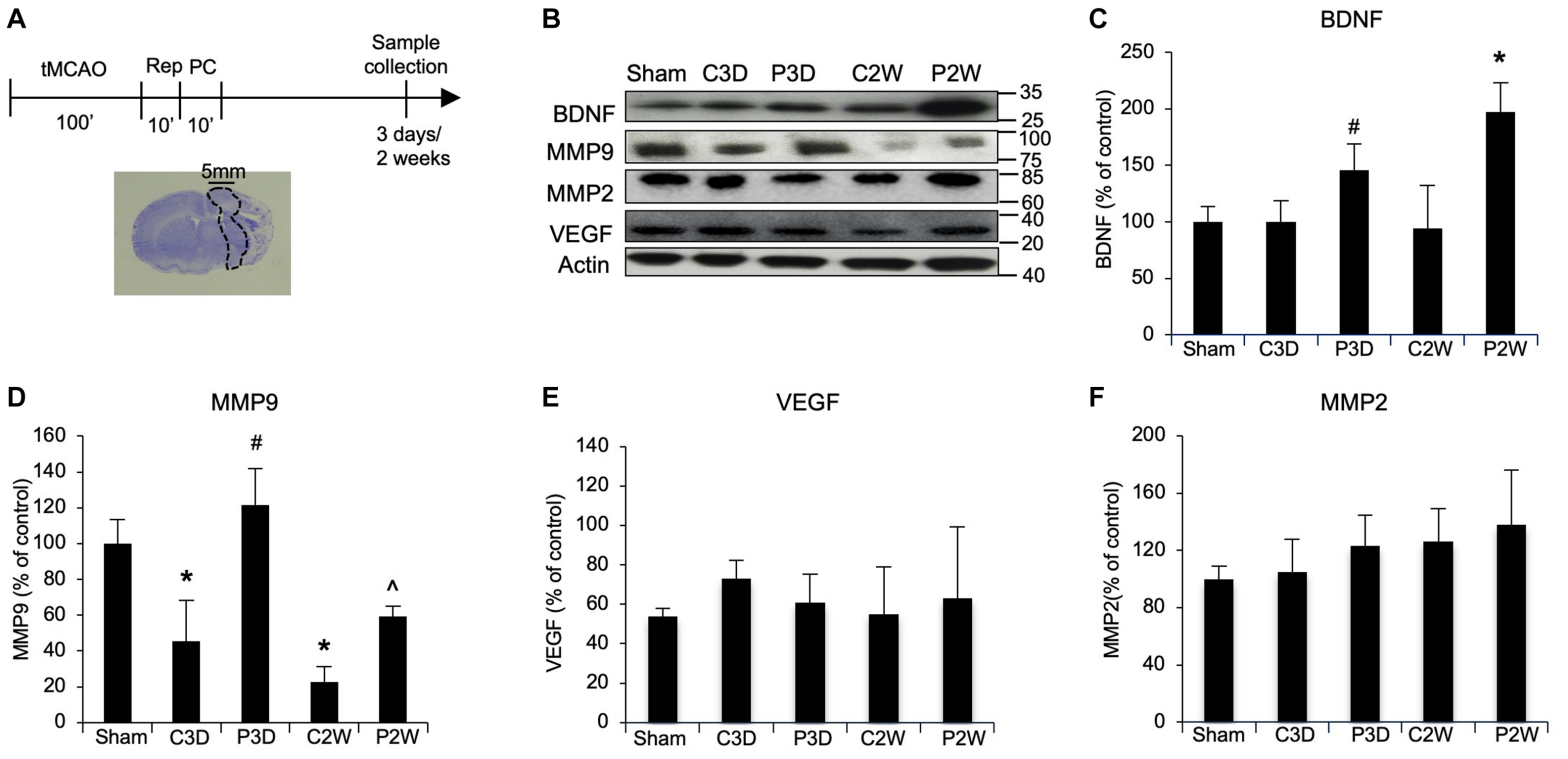
Ischemic postconditioning increases BDNF and MMP9 protein levels after stroke. **(A)** Schematic representation of Ischemic Postconditioning and sample collection. **(B–D)** After MCAO plus postconditioning MMP9 and BDNF protein expression levels increased compared to control animals exposed to tMCAO without postconditioning. **(C–F)** After MCAO plus postconditioning MMP2 and VEGF protein levels did not change when compared with control group. Mean +/− SD. BDNF = *p < 0.05 versus Sham, CTL3D, PostC3D and CTL2W, #*p* < 0.05 versus CTL2W; MMP9 = *p < 0.05 versus Sham, #*p* < 0.05 versus Sham, CTL2W and CTL3D, ^*p* < 0.05 versus Sham, CTL2W and PostC3D (MMP9, MMP2, VEGF *n* = 5).

In order to study the molecular mechanism involved in postconditioning, we analyzed some candidate proteins. Samples were collected from peri-infarct zones, at 3 days and 2 weeks in sham, MCAO and MCAO plus postconditioning groups, and analyzed by western blot analysis (Figure 1A). After MCAO plus postconditioning MMP9 and BDNF protein expression levels increased compared to animals subjected only to MCAO (Figures 1B–D). In contrast, two other astrocyte factors, MMP2 and VEGF, were unchanged (Figures 1E,F).

### BDNF increases in astrocytes after postconditioning

**Figure 2:**
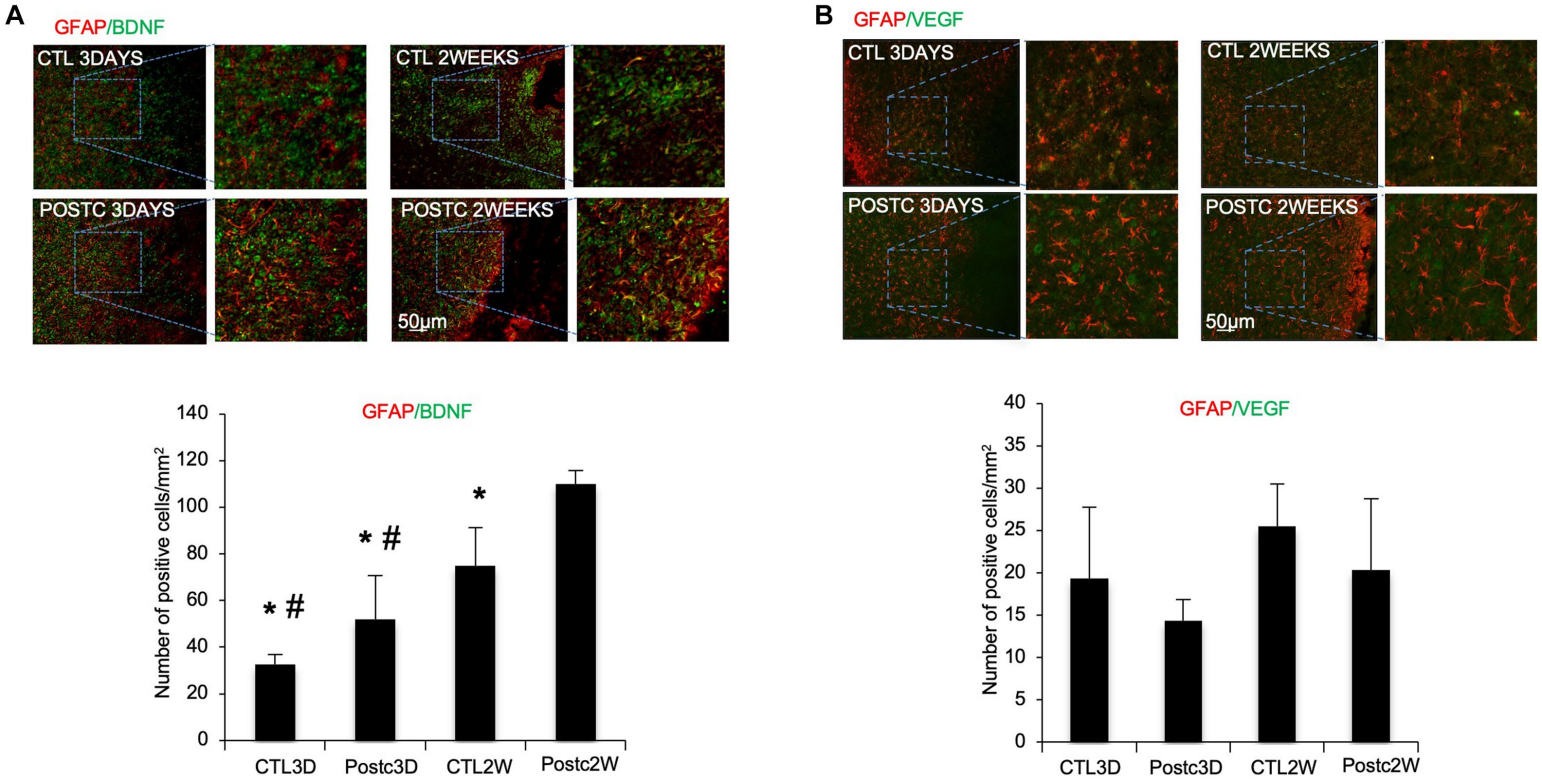
BDNF increases in astrocytes after postconditioning. **(A)** The co-expression of BDNF and GFAP, as astrocytic marker, at 2 weeks post-ischemia was significantly higher after MCAO plus postconditioning, compared with controls. **(B)** Co-expression of VEGF and GFAP, did not change between controls and postconditioning groups. Mean ± SD. *p < 0.05 versus Post2W. #*p* < 0.05 versus CTL2W (*n* = 3).

To investigate which cells were involved in BDNF increase after postconditioning, immunohistochemistry was performed using antibodies against BDNF and GFAP, a marker of activated astrocytes. The co-expression of BDNF and GFAP at 2 weeks post-ischemia was significantly increased after MCAO plus postconditioning, compared with ischemic controls (Figure 2A). We also assessed VEGF, in co-expression with GFAP, since it is known to be the key mediator of angiogenesis, however, no change was observed when postconditioning group was compared to control MCAO group (Figure 2B).

**Figure 3:**
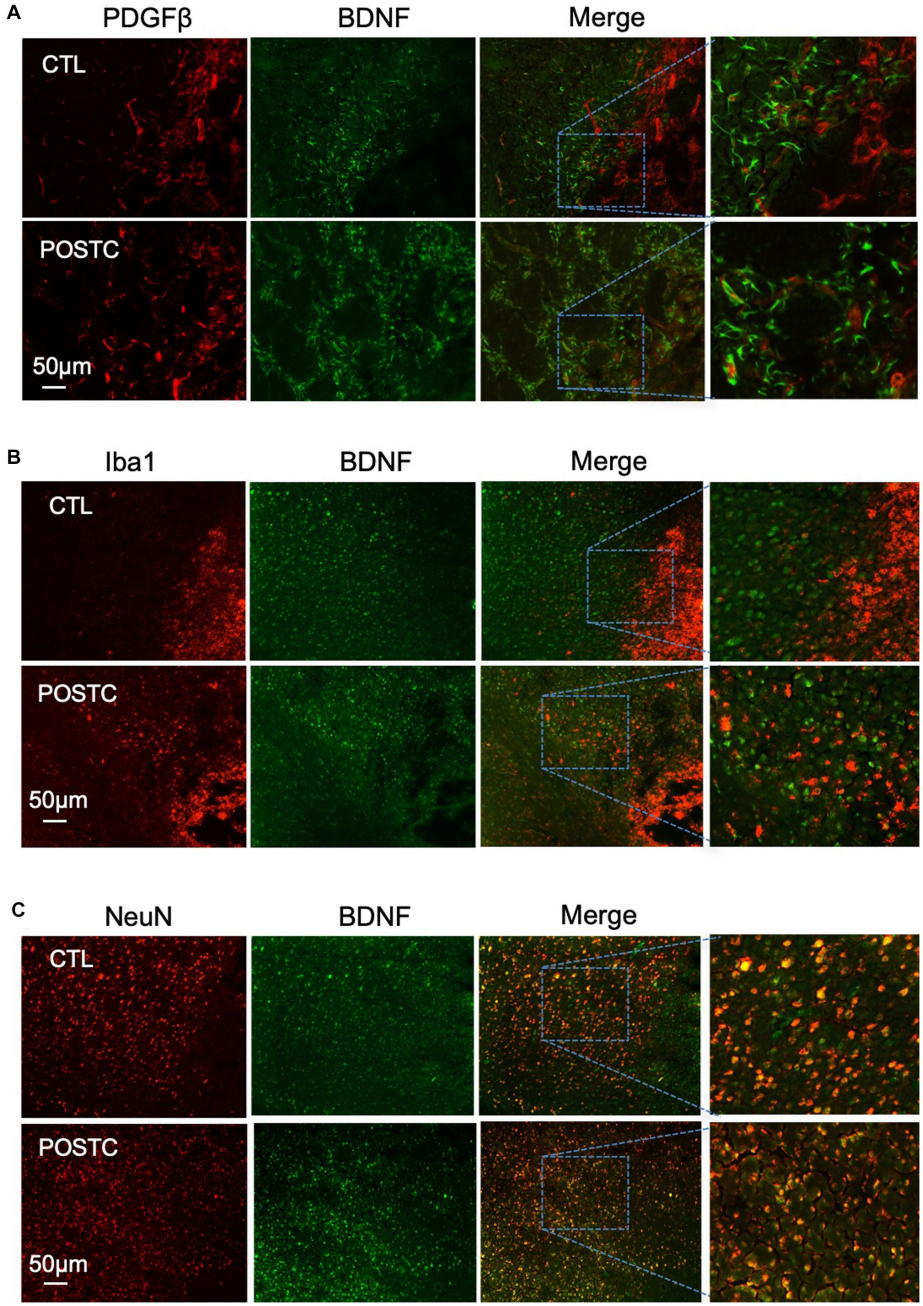
BDNF did not increase in pericytes, microglia and neurons after postconditioning. The co-expression of BDNF with PDGFβ as marker of pericytes **(A)**, or Iba1, as marker of microglia **(B)**, or NeuN, as marker of neurons **(C)**, did not show any difference in postconditioning group compared to stroke alone.

To investigate the role of other cell types in BDNF release after postconditioning, immunohistochemistry was performed using antibodies against PDGFβ as marker of pericytes (Figure 3A), Iba1, as marker of microglia (Figure 3B), or NeuN, as marker of neurons (Figure 3C), co-stained with BDNF. PDGFβ+ cells and Iba1+ cells did not show any prominent release of BDNF. Neurons, as expected, are in part responsible for BDNF release after stroke, indeed the co-expression of BDNF and NeuN seems stronger compared to other the cell types, however no change was observed when comparing BDNF/NeuN double stain in MCAO and MCAO plus postconditioning groups. The contribution of BDNF from neurons needs to be further explored.

### Metabolic astrocyte inhibition prevents postconditioning-induced neuroprotection

**Figure 4:**
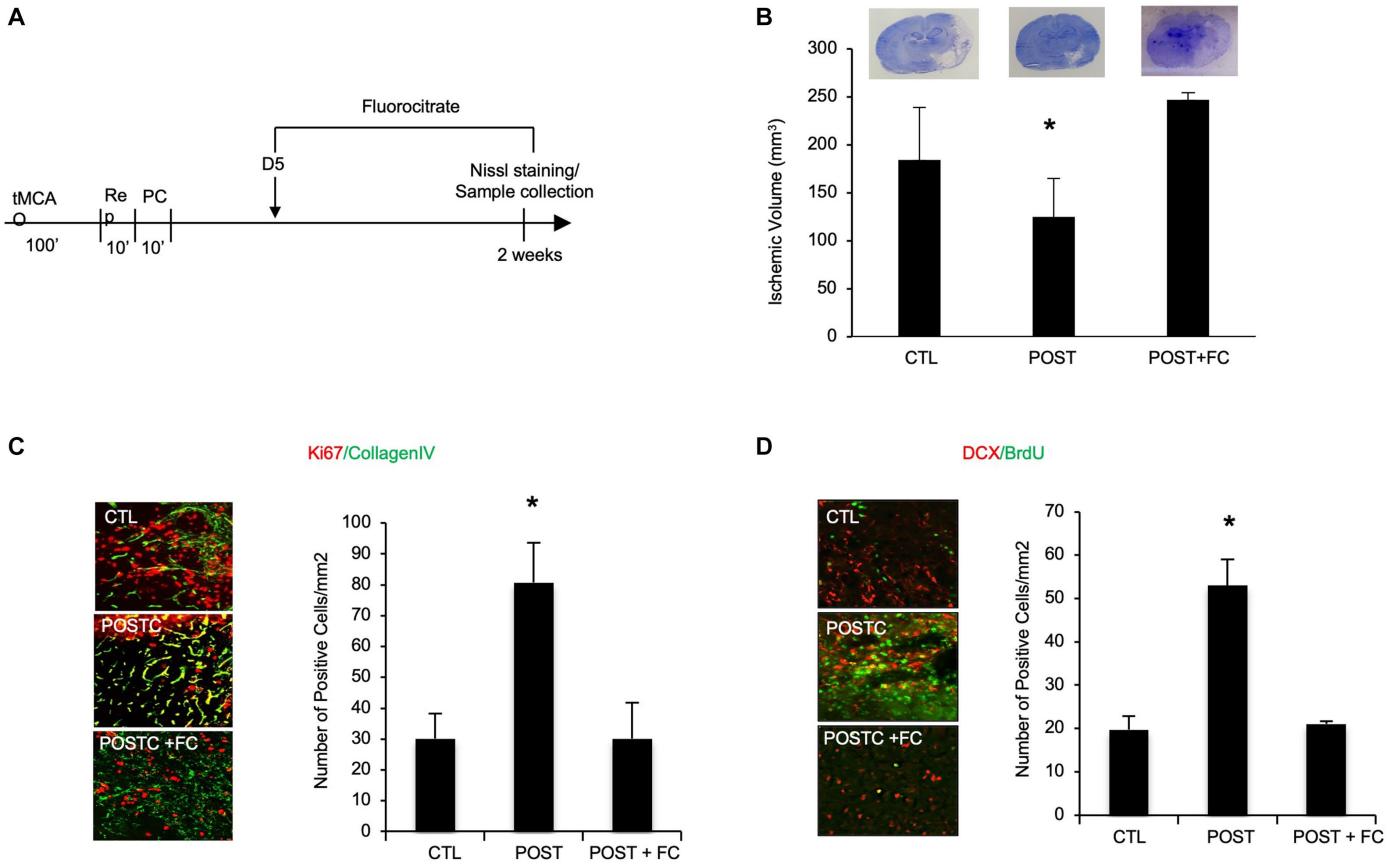
BDNF astrocytes are in part responsible for postconditioning neuroprotection, angiogenesis and neurogenesis. **(A)** Schematic representation of experimental protocol **(B)**. In rats exposed to ischemic postconditioning, infarct volumes were significantly reduced. However, when astrocytes were inhibited by FC (40 nmol) the neuroprotection was prevented. **(C)** The density of collagen-IV-Ki67 microvessels, indicating angiogenesis, was significantly higher in the postconditioning group compared with controls. However, when FC was injected after postconditioning the increase in angiogenesis was no longer observed. **(D)** Immunohistochemistry showed that DCX/BrdU-positive cells, indicating angiogenesis, appeared to be increased in animals subjected to ischemic postconditioning compared to untreated controls. However, the effect was no longer present after FC intraventricular injection. Mean ± SD, *p < 0.05 compared with control and postconditioning plus fluorocitrate (*n* = 3–9).

To demonstrate that during the recovery phase after stroke, astrocytes are involved in neuroprotection induced by postconditioning, they were metabolically inhibited by fluorocitrate (FC) (40 nmol). FC or saline were administrated ICV (intraventricular injection) every other day starting on day 5 after focal cerebral ischemia (Figure 4A). When we compared all the groups for ischemic volume (Nissl staining) we observed that, after astrocyte metabolic inhibition, the neuroprotective effects of postconditioning were in part prevented, indeed ischemic volume was significant higher after flourocitrate plus postconditioning group compared to only postconditioning group (Figure 4B).

Moreover, immunohistochemical analysis showed that the increase in angiogenesis and neurogenesis in MCAO plus postconditioning group was prevented by metabolic astrocyte inhibition. Immunohistochemistry was performed using antibodies against collagen IV and Ki67, for angiogenesis detection, or DCX and BrdU, for neurogenesis detection. The co-expression of collagen IV and Ki67 at 2 weeks post-ischemia was increased after MCAO plus postconditioning compared with ischemic controls, however the increase was no longer detected after fluorocitrate injection. These data suggest that astrocytes might be involved in postconditioning angiogenesis (Figure 4C). The co-expression of DCX and BrdU seemed also increasing after postconditioning, but the effect was no longer present after FC intraventricular injection. These data suggest that astrocytes might be involved in postconditioning neurogenesis (Figure 4D).

## Discussion

Interactions among endothelial cells, glia, neurons and matrix (i.e., the neurovascular unit, NVU) underlie the acute pathophysiology of stroke (Lo and Rosenberg, 2009; Arai et al., 2011; Xing et al., 2012). It is likely that long-term effects of postconditioning are also mediated by cell–cell interactions in the NVU.

In the present study we investigated some of the underlying mechanisms consistent with improvements in poststroke neurogenesis and angiogenesis. Our hypothesis is that ischemic postconditioning can, in part, promote recovery by increasing the capability of the brain to “help itself” via astrocytic production of BDNF.

In a previous study we suggested that the beneficial effects of ischemic postconditioning can be maintained up to 2 weeks post ischemia, in part by promoting neurogenesis and angiogenesis (Esposito et al., 2015). For neural remodeling, astrocytes play an important role by releasing neurotrophic factors such as BDNF. For vascular remodeling, astrocytes may be an important source of pro-angiogenic molecules such as MMP9. Is it possible that ischemic postconditioning improves recovery by augmenting these endogenous processes? Our current data test the hypothesis that post-conditioning affects astrocyte signaling via BDNF in order to promote neurovascular remodeling. Our western blot data show changes in BDNF and MMP9 protein expression levels consistent with the neuroprotective, angiogenic and neurogenetic effect of postconditioning. We also measured VEGF, since it is one of the major factors involved in angiogenesis (Zhang et al., 2000), however, no difference in protein expression was observed after postconditioning. MMP2, together with MMP9, has been shown to be crucial for the “angiogenic switch” that occurs upon the initiation of neurogenesis (Akol et al., 2022), again no change in protein level was observed.

Collectively the results of the present paper suggest that during the recovery phase, postconditioning upregulates the ability of astrocytes to produce BDNF. BDNF, which is a member of the neurotrophin family, is involved in neuroprotection, neurogenesis, neuroplasticity and angiogenesis (Béjot et al., 2011; Grade et al., 2013) and during ischemic preconditioning, BDNF expression is stimulated (Truettner et al., 2002). Our data show that postconditioning also activates this BDNF pathway. Importantly, we also observed MMP9 increase, MMP9 and BDNF responses are related. BDNF can upregulate MMP9 production in rat primary cortical neurons (Kuzniewska et al., 2013), and MMP9 can cleave pro-BDNF converting it to mature BDNF (Mizoguchi et al., 2011). Thus, these coordinated mechanisms may allow postconditioning to amplify and integrate neurogenesis (Jiang et al., 2001; Parent et al., 2002; Ming and Song, 2005) and angiogenesis (Rosell et al., 2013).

While reactive astrocytes were traditionally thought as detrimental, it is now accepted that they can be beneficial under some conditions (Hayakawa et al., 2010; Qu et al., 2010; Barreto et al., 2011). Our data suggest that astrocytes may secrete BDNF and MMP9, which promote neuron survival, synaptic plasticity and angiogenic remodeling during stroke recovery. Indeed, not only the expression of BDNF seems specifically higher in astrocytes, by immunostaining analysis, but by using an astrocyte inhibitor we observed that the neuroprotective effect together with angiogenesis and neurogenesis were partially prevented.

Hence, the ability of postconditioning to amplify these endogenous mechanisms in the neurovascular unit should lead to novel ways to promote stroke recovery.

Nevertheless, there are several issues that should require further investigation. (1) What about other astrocyte factors? Our data point only to BDNF and MMP9, however, other factors such as FGF, HMGB1 and SDF-1 might be released by astrocytes and contribute to the neuroprotective mechanism induced by postconditioning. (2) How can we be sure benefits will disappear after even longer times? Indeed, overall infarcts tend to be smaller over time as brain remodels and shrinks. However previous studies have already showed that ischemic postconditioning neuroprotection lasts up to 1 month (Ren et al., 2008). Although in the present paper, postconditioning was found to be protective at 3 days and 2 weeks, studies exploring longer effect are needed. (3) What about other types of glia? We analyzed the interaction between astrocytes and neurons after postconditioning. Certainly, after stroke, dysfunctions are manifested at the level of cell–cell signaling between neuronal, glial and vascular elements (Arai and Lo, 2010; Ji et al., 2013). Probably a vascular or endothelial event due to reperfusion is also involved. We believe that all the NVU components may ultimately be involved in the benefits of postconditioning, although our data suggest that astrocytes may comprise a key rate-limiting step.

From a clinical perspective, ischemic postconditioning may be attractive as it can be translatable (Vinciguerra et al., 2018), especially for patients subjected to surgery and to endovascular therapy associated with blood vessel occlusion and revascularization. However, most studies so far are focused at 24 h. Additional investigation of postconditioning effects in a chronic phase after stroke may be desirable. Our work aims to define some of the molecular mechanisms responsible of postconditioning neuroprotection during the recovery phase after stroke.

## Data availability statement

The original contributions presented in the study are included in the article/Supplementary material, further inquiries can be directed to the corresponding authors.

## Ethics statement

The animal study was approved by Massachusetts General Hospital Institutional Animal Care and Use Committee in accordance with the National Institute of Health Guide for the Care and Use of Laboratory Animals. The study was conducted in accordance with the local legislation and institutional requirements.

## Author contributions

EE: Conceptualization, Data curation, Writing – original draft. EL: Methodology, Writing – review & editing. OC: Conceptualization, Writing – review & editing. EHL: Conceptualization, Supervision, Writing – review & editing. KH: Supervision, Writing – review & editing. GP: Conceptualization, Writing – review & editing.

## Funding

The author(s) declare financial support was received for the research, authorship, and/or publication of this article. This work was supported in part by grants from the National Institutes of Health, and the Rappaport Foundation.

## Conflict of interest

The authors declare that the research was conducted in the absence of any commercial or financial relationships that could be construed as a potential conflict of interest.

The author(s) declared that they were an editorial board member of Frontiers, at the time of submission. This had no impact on the peer review process and the final decision.

## Publisher’s note

All claims expressed in this article are solely those of the authors and do not necessarily represent those of their affiliated organizations, or those of the publisher, the editors and the reviewers. Any product that may be evaluated in this article, or claim that may be made by its manufacturer, is not guaranteed or endorsed by the publisher.

## Abbreviations

BBB: blood brain barrier
CNS: central nervous system
NVU: neurovascular unit
BDNF: brain-derived neurotrophic factor
MCA: middle cerebral artery
PBS: phosphate buffered saline
tMCAO: transient middle cerebral artery occlusion
MMP: matrix metalloproteinases
VEGF: vascular endothelial growth factor
GFAP: glial fibrillary acidic protein
PDGFβ: platelet derived growth factor beta receptor
NeuN: neuronal nuclear protein
Iba1: ionized calcium-binding adaptor molecule 1
FC: fluorocitrate
ICV: intraventricular injection
DCX: doublecortin
BrdU: bromodeoxyuridine
5D: 5 days
Rep: reperfusion
PC: postconditioning
CTL3D: controls 3 days
PostC3D: postconditioning 3 days
CTL2W: controls 2 weeks
PostC2W: postconditioning 2 weeks
CTL: controls
POST: postconditioning
POST+FC: postconditioning plus fluorocitrate
